# Book Notices

**Published:** 1882-12-20

**Authors:** 


### *BOOK NOTICES.*

THE PHYSICIANS' VISITING LIST. (Lindsay & Blakiston's) for 1883. Thirty-second year of its publication. Philadelphia: P. Blakiston, Son & Co., successors to Lindsay & Blakiston, 1012 Walnut street. Sold by all Booksellers and Druggists.

This work has a deservedly high reputation, established by long years of use by medical practitioners. It contains much useful and valuable information, besides a well arranged order of blanks for entries and memoranda. Price for 25 patents $1.00, and higher rates for larger number of patents.

ON SLIGHT AILMENTS—THEIR CAUSES, NATURE AND TREATMENT. By Lionel S. Beal, M. D., F. R. S., Prof. of Practice of Medicine at Kings College, London. Second edition, revised, enlarged and illustrated. Philadelphia: P. Blakiston, Son & Co., 1012 Walnut Street, 1882. Cloth, 283 octavo pages. Price, $1.25.

We regard the above as a very useful and valuable book for practitioners.

DISEASES OF THE LIVER, WITH AND WITHOUT JAUNDICE—with the Special application of Physiological Chemistry to their Diagnosis and treatment. By George Harley, M. D., F. R. S., Fellow of the Royal College of Physicians—Corresponding member of the Academy of Sciences of Bavaria; of the Academy of Medicine, of Madrid, and of several Continental Medical Societies. Formerly President of the Parisian Medical Society, Physician to University College Hospital, and Professor in University College, London. Illustrated by colored plates and wood engravings. Philadelphia: P. Blakiston, Son & Co., 1012 Walnut Street, 1882.

The above work contains 750 octavo pages, and is neatly gotten up. It is printed simultaneously with the London edition, and contains the full text and original illustrations. Although a great deal has been written upon the Liver and its affections, there remains much obscurity as to the real nature and treatment of this class of diseases. Dr. Harley has here brought to bear his long and extensive experience in the study and treatment of hepatic affections, with the special application of Physiological Chemistry to diagnosis and treatment.

The work is ably written and contains a large amount of Clinical and Scientific information, not heretofore thrown together in a single volume, with new facts and theories of modern scientific advancement. We commend this work to the Profession as eminently worthy of study, and as one which should be in the library of every physician.

